# Temporal and spatial characterization of keratinocytes supporting orf virus replication

**DOI:** 10.3389/fcimb.2024.1486778

**Published:** 2025-01-31

**Authors:** Byung-Joon Seung, Sushil Khatiwada, Daniel L. Rock, Gustavo Delhon

**Affiliations:** ^1^ Department of Pathobiology, College of Veterinary Medicine, University of Illinois at Urbana-Champaign, Urbana, IL, United States; ^2^ School of Veterinary Medicine and Biomedical Sciences, and Nebraska Center for Virology, University of Nebraska-Lincoln, Lincoln, NE, United States

**Keywords:** orf virus, pathogenesis, keratinocytes, full thickness wound skin model, sheep, *in situ* hybridization (ISH)

## Abstract

Reflecting their tropism for keratinocytes, most poxviruses that infect vertebrates replicate to high titers and cause pathology in the skin. Keratinocytes, the main cells of the epidermis, are found in different stages of a differentiation program that produces the critical barrier against environmental damage. While systemic poxviruses (e.g. smallpox virus, sheeppox virus) also infect other cell types, the parapoxvirus orf virus (ORFV), which causes localized infections in sheep and goats, has not been shown to replicate in cells other than keratinocytes. Notably, ORFV infection only occurs after or concomitant with epidermal damage and the subsequent healing response and shows unexplained delayed virus replication in an uncharacterized keratinocyte subpopulation. Using *in situ* hybridization, immunohistochemistry, confocal microscopy, qPCR, and a full-thickness wound/infection model in sheep, the natural host, we show that during an initial 2-day eclipse phase viral transcription and viral DNA replication are not detected. Between days 2 and 3 pi, viral transcription is first detected in keratinocytes of the stratum granulosum and upper stratum spinosum in the proliferative zone at the wound margin. These cells are positive for cytokeratin 10, a suprabasal marker; cytokeratin 6, a protein induced during early repair responses; stratum granulosum markers filaggrin and loricrin; and negative for the nuclear proliferation marker Ki-67 and cytokeratin 14, a basal cell marker. This marker profile suggests that keratinocytes supportive of viral replication are engaged in advanced keratinocyte differentiation rather than proliferation.

## 1 Introduction

*Parapoxvirus orf* or orf virus (ORFV), the type member of the genus *Parapoxvirus* (PPV) of the *Poxviridae*, causes orf or ecthyma contagiosum, a highly contagious disease of sheep and goats worldwide. Orf is characterized by self-limited lesions in the mucocutaneous transitions of the mouth, nostrils, and mammary glands, and in the oral mucosa (Robinson and Balassu, 1981; Haig and Mercer, 1998). In non-complicated cases, clinical resolution is achieved in 4 to 6 weeks. Orf transmission occurs by direct contact with acutely infected animals, shed scabs, contaminated fomites, and, potentially, subclinically infected animals (Nettleton et al., 1996; Ma et al., 2022). Orf is a zoonotic disease affecting humans in close contact with infected animals (Caravaglio and Khachemoune, 2017).

ORFV is highly epitheliotropic, and keratinocytes and their ontogenetically related counterparts in the oral mucosa are the most important cell type supporting ORFV replication *in vivo* (McKeever et al., 1988). The virus penetrates the epidermis through small skin/oral breaks. Skin scarification, a procedure that damages the interfollicular and proximal follicular epidermis while sparing deeper tissues, followed by a topical application of virus inoculum have been traditionally used to efficiently infect experimental animals and for vaccination with ORFV (Clover, 1928). This and clinical observation of field cases have suggested that skin damage and the subsequent wound healing response (WHR) are indeed required for efficient ORFV infection of the skin (Boughton and Hardy, 1934; Robinson and Balassu, 1981; Watt, 1983; Savory et al., 2000), a notion that is further supported by failure to establish ORFV infection following inoculation by the intradermal (ID) and subcutaneous (SC) routes (Boughton and Hardy, 1934; McKeever et al., 1988), and by microscopic observations linking virus replication to epidermal healing processes (Jenkinson et al., 1990b).

The pathology of natural ORFV infections is characterized by the development of highly vascularized, often proliferative lesions around virus entry sites. Lesions evolve through erythema, papule, pustule, and, for the tegument, scab deposition stages, with those in the mucosa becoming more papillomatous and ulcerative (Robinson and Balassu, 1981; McElroy and Bassett, 2007). Microscopic examination of sheep lesions during natural and experimental orf shows 1) epidermal acanthosis and ballooning degeneration of keratinocytes of the stratum spinosum of the interfollicular and upper follicular epidermis starting on days 2 to 5 post-infection (pi); 2) limited lateral and basal epidermal spread of the infection; 3) dermal inflammatory cell infiltration and neovascularization; 4) vesicle and pustule formation followed by pustule rupture and crust formation; 5) papillomatous development of rete pegs and dermal papillae; and 6) crust shedding, epidermal regeneration, and resolution of dermal changes, with the full process taking 40 to 60 days (Aynaud, 1923; Clover, 1928; Selbie, 1945; Wheeler and Cawley, 1956; Kluge et al., 1972; McKeever et al., 1988; Jenkinson et al., 1990a). Infected keratinocytes undergo ballooning degeneration and, occasionally, contain acidophilic inclusion bodies in the cytoplasm. ORFV antigen in infected keratinocytes is first detected between days 2 and 3 pi and remains detectable for approximately 10-12 days (McKeever et al., 1988; Jenkinson et al., 1990a).

While orf lesion progression has been extensively examined in the natural host, important questions regarding disease pathogenesis remain unresolved, including the identity of the keratinocytes supporting ORFV replication, the state of the virus during the first 2 days pi, and the potential of the virus to persist asymptomatically in the host (Osman, 1976; Jenkinson et al., 1990a; Ma et al., 2022). Answers to these questions are particularly challenging as the progression of infection overlaps with wound healing processes. To examine these issues, we have used a full-thickness wound (FTW) model, a type of excisional wound that comprises the epidermis, the basal membrane, and the dermis, and that can be easily produced with a sterile punch. The rationale behind this modification is that the FTW 1) resembles the wound caused by prickly plants and hard stubbles often associated with natural orf infection (Boughton and Hardy, 1934; Robinson and Balassu, 1981; Watt, 1983); 2) when implemented by punch biopsies, it is easily produced and replicated (Wilhelm et al., 2017); 3) it has been extensively used and characterized at the cellular and molecular levels as a wound model to study mammalian skin repair (Langton et al., 2008; Aragona et al., 2017; Park et al., 2017); and 4) it allows for better control of virus dosage than topical inoculation on scarified skin. Using FTW followed by ORFV inoculation it was found that 1) during the initial 2-day eclipse phase, there is no sign of viral DNA replication and viral transcription at inoculation sites; 2) viral transcription is detected first in the proliferative zone at the wound margin between 2 and 3 dpi; 3) keratinocytes supporting viral transcription and replication are localized in the upper stratum spinosum and in the stratum granulosum of the healing epidermis; and 4) the involved keratinocytes are positive for cytokeratin 10, cytokeratin 6, loricrin, and filaggrin markers, and negative for the nuclear proliferation marker Ki-67 and cytokeratin 14.

## 2 Materials and methods

### 2.1 Cells and medium

Primary ovine fetal turbinate (OFTu) cells were used for virus propagation and recombinant virus production. OFTu cells were maintained in minimal essential medium (MEM) (Corning, Glendale, AZ) supplemented with 10% fetal bovine serum (FBS) (Atlanta Biologicals, Flowery Branch, GA), 2 mM L-glutamine, gentamicin (50 μg/ml), penicillin (100 IU/ml), and streptomycin (100 μg/ml). Cells were incubated at 37 °C with 5% CO2.

### 2.2 Construction of virus OV-IA82-RV120^3XFlag^

OV-IA82-Δ120 virus was used as the parental virus to construct a revertant virus for pathogenesis studies, OV-IA82-RV120^3XFlag^. A recombination cassette was constructed containing the early-late viral gene ORFV120 with a C-terminal 3XFlag, followed by reporter gene red fluorescent protein sequences under the control of vaccinia virus 7.5K early promoter. To promote homologous recombination, these sequences were flanked by ~700 bp of upstream and downstream ORFV120 sequences. Production of OV-IA82-RV120^Flag^ was performed as previously described (Khatiwada et al., 2021). Briefly, OFTu cells were infected with OV-IA82 (MOI 1), transfected with the recombination plasmid, and recombinant viruses were isolated by limiting dilution and plaque assay using fluorescence microscopy. The identity and integrity of the viral DNA sequences were confirmed by PCR and DNA sequencing. OV-IA82-RV120^Flag^ exhibited wild type virus virulence characteristics in the infected sheep.

### 2.3 Animal inoculations and sampling

**Figure 1:**
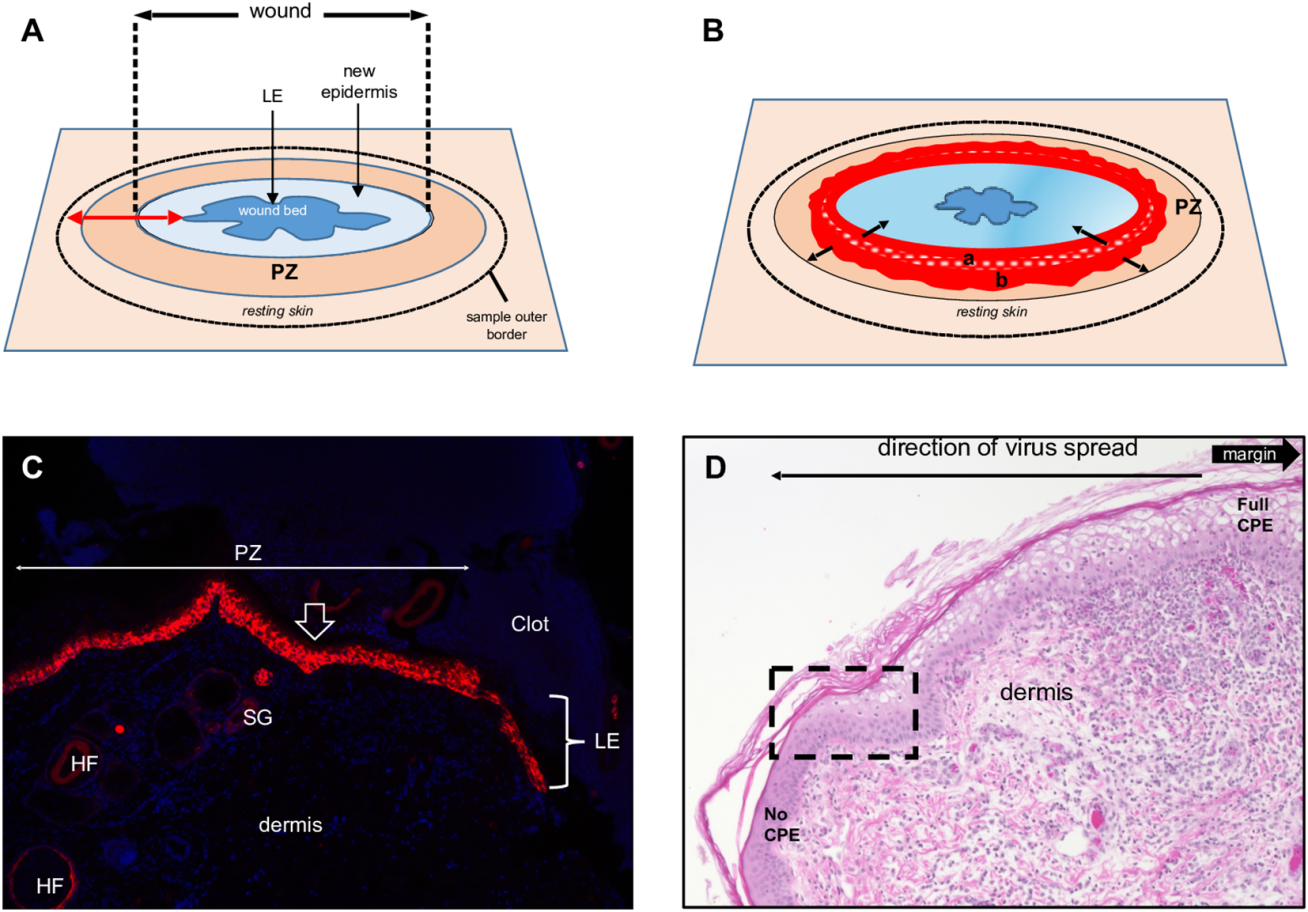
Full-thickness wound model of ORFV infection in sheep. **(A)** Schematic of a 3-to-5-day full- thickness incisional wound showing the leading edge (LE), new epidermis, proliferative zone (PZ), and the position of the biopsy sample outer border. **(B)** Virus-infected wound (5 dpi). The red area is the virus-infected epidermis; inner subarea **a** has initially infected cells, outer subarea **b** has more recently infected cells; black arrows show infection spread direction. **(C)** Uninfected wound (3 dpw) showing the wound margin (open arrow), PZ, and LE. Red, RNA-ISH for CK-14; blue, DAPI, X100. HF, hair follicles; SG, sebaceous gland. **(D)** Infected skin, 5 dpi. Note the gradual change of the epidermal PZ from full keratinocyte ballooning (right, full CPE) to intact epidermis (left, no CPE); rectangle, area targeted in this work to characterize newly infected keratinocytes. H/E X200.

For microscopic examination of ORFV-infected skin, 70-90 lb lambs were anesthetized with ketamine/midazolam, and full-thickness wounds (FTW) were performed via 3mm punch biopsies in the skin of the inner side of the thighs. Then, 20 ul of a virus stock containing 10^7.5^ TCID_50_/ml of OV-IA82-RV120^3XFlag^ (infected group, sheep # 12, 21, 30, 104, and 374) or sterile PBS (uninfected group; sheep # 34 and 82) were deposited in the wound bed. To monitor disease progression in the lip, the sheep were additionally inoculated with either OV-IA82-RV120^3XFlag^ (0.5 ml of a 10^7.5^ TCID50/ml inoculum) or PBS in a scarified area of the ventral lip near the left labial commissure. At 24, 48, 60, 72, 96, 120, 144, and 168 h pi, a second, 6mm wide punch was performed around pre-anesthetized (2% lidocaine) inoculation sites to collect the entire wound and surrounding normal skin (**Figure 1A**). Five samples from different infected sheep were collected for each time point except for 24h and 60h (three samples each). For uninfected controls, two samples per time point were collected. Samples were fixed and processed for standard histology. For PCR detection of viral nucleic acids at inoculation sites, six additional sheep (#33, 190, 196, 204, 240, and 533) were infected and sampled as above, with the exclusion of a 60 hpi sample and the inclusion of a 10 dpi sample. PCR samples were frozen at -80C until processing.

### 2.4 Histology

Tissue samples were fixed in 10% neutral buffered formalin and embedded in paraffin. Serial tissue sections encompassing the entire wound were obtained perpendicular to the skin surface and mounted on SuperFrost Plus Slides (Fisher Scientific, Pittsburgh, PA). One slide from each series was stained with hematoxylin and eosin (H&E) to evaluate and determine the optimal level and number of additional sections.

### 2.5 Immunohistochemistry

The spatial distribution of the late epidermal differentiation markers filaggrin and loricrin, the proliferation marker Ki-67, and cytokeratins (CKs) 6 (CK-6, repair keratinocytes), 10 (CK-10, stratum spinosum keratinocytes), and 14 (CK-14, stratum basale keratinocytes) was investigated by immunohistochemistry (IHC). Sections were deparaffinized, rehydrated, and blocked with BLOXALL^®^ Blocking solution (Vector Laboratories, Newark, CA). Heat-induced antigen retrieval was performed using citrate buffer (pH 6.0) in a pressure cooker for 15 minutes. After blocking with 5% normal goat serum in PBS, sections were incubated with primary antibodies at 4°C overnight (see antibodies and dilutions in **Supplementary Table 1**). The ImmPRESS^®^ Goat Anti-Mouse IgG polymer kit (Vector Laboratories; #MP-7452) and the DAB substrate kit (Vector Laboratories; #SK-4100) were used for immunolabelling and visualization, followed by counterstaining with Gill’s hematoxylin. Images were captured using a Keyence BZ-X800 microscope (Keyence, Japan) or NanoZoomer 2.0-HT slide scanner (Hamamatsu, Japan).

### 2.6 Immunofluorescence

To detect OV-IA82-RV120^3XFlag^ in tissues, immunofluorescence was performed. Sections were deparaffinized, rehydrated, permeabilized with 0.1% triton X-100, and subjected to heat-induced antigen retrieval with citrate buffer (pH 6.0) in a pressure cooker for 15 min. After blocking with 5% BSA in PBS, sections were incubated overnight at 4°C with primary antibody against FLAG, followed by Alexa fluor 488 labeled secondary antibody. Sections were mounted with VECTASHIELD Vibrance Antifade Mounting Medium with DAPI (Vector Laboratories; #H-1800-10). Images were acquired using a Nikon A1 confocal microscope (Nikon, Japan).

### 2.7 RNA *in situ* hybridization (chromogenic assay)

Chromogenic visualization of viral replication was performed using RNAscope 2.5 HD brown assay (Advanced Cell Diagnostics, Newark, CA; #322300). To detect ORFV OV-IA82 DNA and RNA, three viral gene targets were selected: immediate early gene ORFV113, early gene ORFV064, and late gene ORFV011 (Joshi et al., 2023). Anti-sense probes for these genes were as follows (targeted genomic segments indicated in parentheses): ORFV113 probe (ACD; #570061), ORFV064 probe (ACD; #1279031-C1), and ORFV011 (ACD; #1180021-C1). Oa-POLR2A, a probe targeting ovine RNA Pol II A subunit (ACD; #615171) and DapB, a probe targeting bacterial dihydrodipicolinate reductase gene (ACD; #310043) served as the positive and negative controls, respectively. All procedures were manually carried out following the manufacturer’s instructions. Briefly, 5-μm-thick sample sections were baked at 60°C for 1 h, deparaffinized in xylene and dehydrated in ethanol. Sections were treated with RNAscope Hydrogen Peroxide reagent (ACD; #322330) for 10 min, followed by antigen retrieval using RNAscope Target Retrieval Reagent (ACD; #322000) at 93°C to 98°C for 15 min. Sections were then incubated with RNAscope Protease Plus Reagent (ACD; #322330) for 30 min at 40°C. To detect viral DNA only, sections were treated with 5mg/ml RNase A (Qiagen; #19101) in PBS. For viral RNA only, sections were treated first with DNase-I (Sigma; #D5319-500UG) diluted 1:50 in 1x DNase buffer, followed by Protease Plus treatment. Probes were hybridized for 2 h at 40°C, followed by signal amplification and detection using DAB. Counterstaining was performed with 50% Gill’s hematoxylin, and images were acquired using a Keyence BZ-X800 microscope.

### 2.8 RNA *in situ* hybridization (fluorescence assay) and immunofluorescence

To characterize the keratinocytes supporting ORFV replication, multiplex RNA-ISH and IF were performed using an RNAscope Multiplex Fluorescent V2 Kit (ACD; #323100) combined with an RNA-Protein Co-detection Ancillary Kit (ACD; #323180). Tissue sections were deparaffinized, dehydrated, and treated with hydrogen peroxide, and antigens were retrieved with 1X Co-Detection Target Retrieval Reagents (ACD; #323165) at 93°C to 98°C for 15 min. Sections were incubated with primary antibodies (CK-10/CK-14, Ki67/CK6, Loricrin/Filaggrin) diluted in Co-Detection Antibody Diluent (ACD; #323160) overnight at 4°C. After washing, sections were post-fixed in 10% neutral buffered formalin, treated with RNAscope protease Plus, and hybridized with probes targeting ORFV064, ORFV113, or ORFV011. The positive and negative controls used were Oa-POLR2A and DapB, respectively. Signal amplification was performed, followed by HRP-C1 and TSA Vivid Fluorophore 520 (Tocris Bioscience, UK; #7523). After RNA-ISH signal development, sections were incubated with the appropriate fluorophore-conjugated secondary antibody diluted with Co-Detection Antibody Diluent for 30 min at room temperature (RT). Antibodies and their dilutions are shown in **Supplementary Table 1**. Slides were counterstained with DAPI and mounted with ProLong Gold Antifade Mountant (Thermo Fisher Scientific; #P36930). Images were acquired using a Nikon A1 confocal or Keyence BZ-X800 microscope.

### 2.9 RNA *in situ* hybridization for CK-14 RNA (KRT14)

To detect CK-14 RNA and viral RNAs, tissue sections were processed as described above with RNAscope Target Retrieval Reagent (ACD, # 322000) for antigen retrieval, without combining with an RNA-Protein Co-detection Ancillary Kit. Sections were hybridized with ORFV probes and Oa-KRT14-O1-C2 (ACD, #1573381-C2). Signals were developed using TSA Vivid Fluorophore 520 (for ORFV) and 570 (for CK-14) (Tocris Bioscience, #7523, #7526). All other steps were as previously described.

### 2.10 TaqMan real-time PCR

Skin samples were collected from six sheep (#33, 190, 196, 204, 240, and 533) at days 1 and 2 pi, from four sheep (#33, 190, 204, and 533) at days 3 and 4 pi, and from two sheep (#190 and 204) at days 5, 6, 7, and 10 pi, and DNA was extracted using a DNeasy Blood & Tissue Kit (Qiagen, Germany; #69504) following the manufacturer’s recommendations. DNA was analyzed for viral DNA using TaqMan real-time PCR with primers and probes for genes ORFV118, ORFV119, and ORFV121 (**Supplementary Table 2**). The PCR was performed on a QuantStudio 3 instrument (Applied Biosystems, Waltham, MA) with 20 μl reactions containing 10 μl TaqMan Fast Advanced Master Mix (Applied Biosystems; #4444557), 1 μl each specific primer (500 nM) and probe (250 nM) (synthesized by IDT, Newark, NJ), 7 μl nuclease-free water, and 2 μl template DNA. Reactions were run in triplicate with cycling conditions of 2 min at 50°C and 10 min at 95°C, followed by 40 cycles of 15 s at 95°C and 1 min at 60°C. DNA from normal skin was used as a negative control. Average CT values were used to generate a virus growth curve. One-way ANOVA and Tukey’s HSD *post-hoc* analysis were performed to identify significant differences among time points using GraphPad Prism version 9 (GraphPad Software, La Jolla, CA).

### 2.11 Transmission electron microscopy

Two sheep were infected (right leg) or mock- infected (left leg) as described above, and skin samples were collected at various times pi, cut into 1mm pieces, and fixed in 2% glutaraldehyde and 2% paraformaldehyde in 0.1M cacodylate buffer (pH 7.2) for 2 h at RT. Post-fixation was performed with 1% osmium tetroxide for 90-120 min at RT. The samples were then dehydrated through a graded ethanol series, transferred in a series of ethanol:Spurr resin mixtures, embedded in 100% Spurr, and polymerized at 65°C for 24-36 h. Ultrathin sections (70-80 nm) were obtained with a Leica EM UC7 Ultramicrotome. Sections were collected on copper grids and stained with 2% uranyl acetate and Reynolds’ lead citrate to improve the contrast and examined using Hitachi HT7800 TEM at 80 KV. Digital micrographs were collected using an AMT NanoSprint1200 CMOS camera.

## 3 Results

### 3.1 Full-thickness wound repair in mock-infected control sheep

ORFV replication *in vivo* has been linked to epidermal repair processes (Jenkinson et al., 1990b), yet questions regarding the timing of infection and identity of the targeted keratinocyte subpopulations remain unanswered. Most ORFV pathogenesis studies in the natural host have relied on the experimental infection of scarified skin. Issues of reproducibility, repair model characterization, and virus dosage accuracy associated with the scarified skin wound (SW) model led us to explore the suitability of the full-thickness excisional wound (FTW) as a model to study ORFV infection (Masson-Meyers et al., 2020). We initially examined skin changes following FTW in uninfected sheep. Three mm punches were performed in the inner side of the thighs of sheep # 34 and # 82, followed by the addition of PBS to the wound bed. Six mm punches were then daily collected around the original punches on days 1-7 post-wound (pw), and tissue samples were processed for standard histology and IHC.

**Figure 2:**
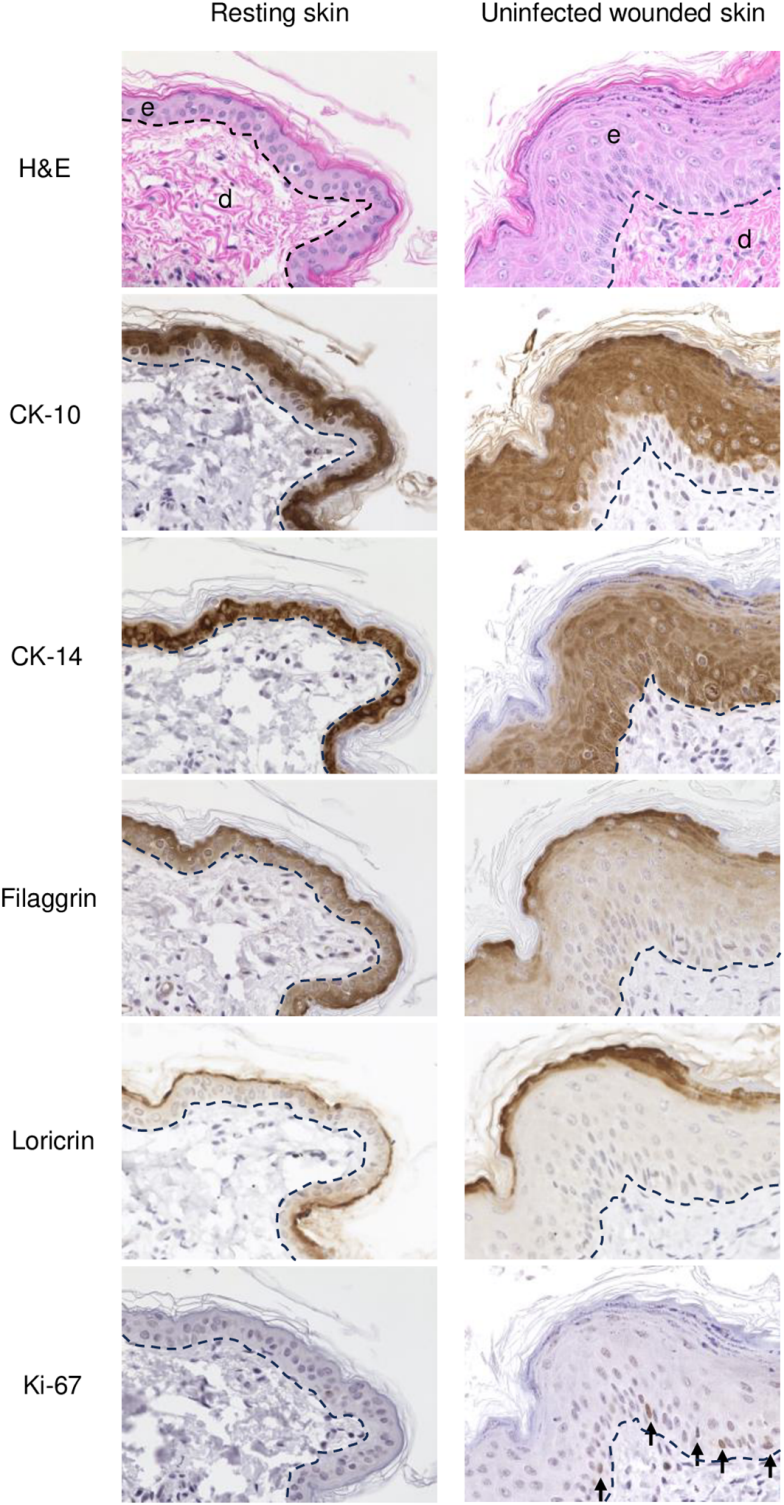
IHC of normal (resting) and wounded sheep skin. Left column, resting skin. Right column, uninfected wounded skin (PZ) on 3 dpw. Top, e, epidermis; d, dermis; H/E. IHC panels show staining for cytokeratin 10 (CK-10), cytokeratin 14 (CK-14), filaggrin, loricrin, and Ki-67 (arrows, positive nuclear labeling). The dashed lines indicate the position of the basement membrane. X400.

Overall, the microscopic features of the WHR resembled those previously described for an FTW model in humans (Patel et al., 2006). Early neutrophil infiltration of the denuded dermis was replaced by mononuclear infiltration by day 3 pw. Reactive epidermal hyperplasia (i.e. acanthosis) produced a proliferative zone (PZ) around the wound margin that resulted in a 4 to 7-fold increase in epidermal thickness by day 3-5 pw (**Figures 1A, C**; **Figure 2**, right panels). PZ keratinocytes were stained by antibodies against CK-10 suprabasally, CK-14 and proliferation marker Ki-67 basally and suprabasally, and filaggrin and loricrin apically (**Figure 2**). This IHC profile was similar to the one found in resting (unwounded) skin, except the latter showed minimal to no reactivity to Ki-67, and staining for cytokeratins and filaggrin was more intense than in the wounded skin PZ (**Figure 2**, compare left and right panels). In addition, while staining for CK-14 was restricted to the basal layer in the normal epidermis, labeling expanded to most suprabasal layers in the PZ as was also shown in wounded human skin (Aden et al., 2008; Mendoza-Garcia et al., 2015). This effect, however, was less pronounced when CK-14 was stained with the same antibody but using immunofluorescence, or when the tissue was probed for CK-14 transcripts (**Figure 1C**). Starting on days 1-2 pw, keratinocytes at the wound margin migrated centripetally over the wound bed, forming a leading edge (LE) that was positive for CK-14 and injury-induced CK-6, and negative for CK-10, Ki-67, loricrin, and filaggrin, confirming that the migratory front is not engaged in proliferation nor differentiation (**Supplementary Figures 1A, B**; (Patel et al., 2006)). The appearance of CK-10 staining in cells left behind in the LE indicated that keratinocytes started to differentiate by day 5 pw. By days 6 to 7 pw, the wound was fully covered by a new epidermis, with little to no evidence of the LE remaining. The new epidermis was 4 to 5-fold thicker than the resting epidermis around the wound, exhibited the four-strata typical of unwounded skin, and showed a similar CK-10/CK-14/loricrin/filaggrin staining pattern as observed for the PZ on day 3 pw (**Figure 2**, right column). Epidermal rete peg development and inflammatory infiltration of the underlying dermis were negligible by day 7 pw (2 to 5 pegs per 10 sections), and no indication of the *de novo* formation of hair follicles and skin glands was observed beneath the wound bed.

### 3.2 ORFV infection of full- thickness incisional skin wounds

Eleven sheep (ID # 12, 21, 30, 33, 104, 190, 196, 204, 240, 374, and 533) were infected with OV-IA82-RV120^3XFlag^, a virus derived from strain OV-IA82 (Delhon et al., 2004) that carries 3Xflag sequences fused to the C-terminus of ORFV120, a non-essential early-late gene. OV-IA82-RV120^3Xflag^ replication *in vitro* and virulence in sheep are indistinguishable from the parental virus. OV-IA82-RV120^3Xflag^ was inoculated in the inner side of the thighs using the FTW approach, and tissue samples for microscopy (sheep # 12, 21, 30, 104, and 374) and qPCR (sheep # 33, 190, 196, 204, 240, and 533) were collected at various times pi. As a clinical control for orf, the lower lip was scarified near the left labial commissure and topically inoculated with the virus. All sheep showed typical orf lesions in the lip starting at day 3 pi, with gross changes that were comparable to those at the thigh inoculation sites (**Supplementary Figure 2**).

**Figure 3:**
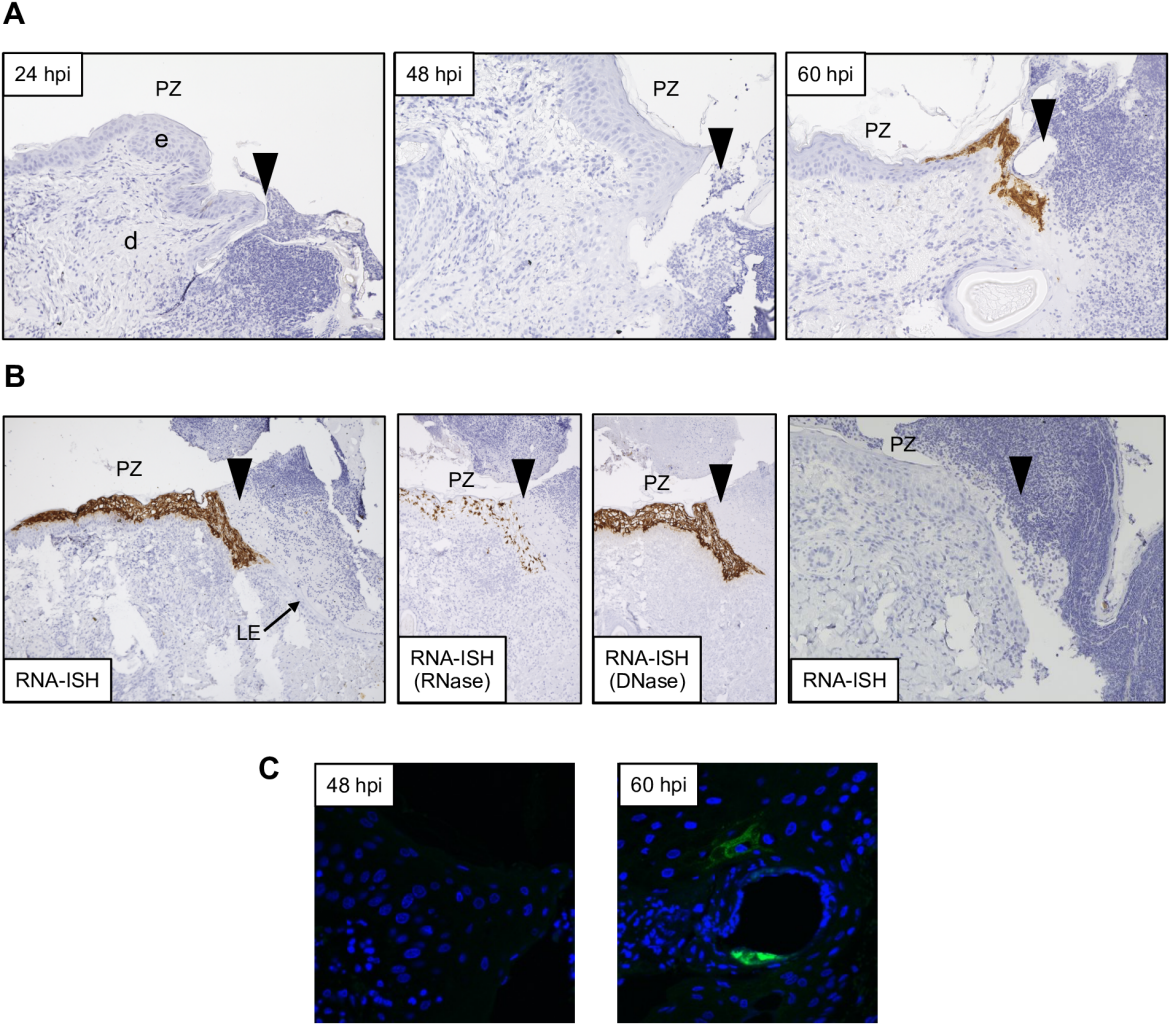
Detection of ORFV transcripts and antigen at skin inoculation sites. Sheep were inoculated with OV-IA82-RV120^3XFlag^ in the inner side of the thighs (see M&M), and tissue samples collected at various times pi, fixed and processed for ISH (A and B; ORFV113 probe) or IF **(C)**. Arrowheads in A and B indicate left margins of sheep #21 wounds; results were similar for probes ORFV113 and ORFV011. **(A)** RNA-ISH images of the PZ at 24, 48, and 60 hpi; X200. **(B)** Left panel, RNA-ISH of the PZ at 4 dpi; central panels, RNase and DNase controls, X100. Right panel, RNA-ISH in uninfected sheep PZ 4 dpw (sheep #82), X200. **(C)** Confocal microscopy of panel A samples at 48 hpi and 60 hpi with anti-Flag antibody and DAPI. X600. e, epidermis; d, dermis; PZ, proliferative zone of the epidermis; LE, leading edge.

**Figure 4:**
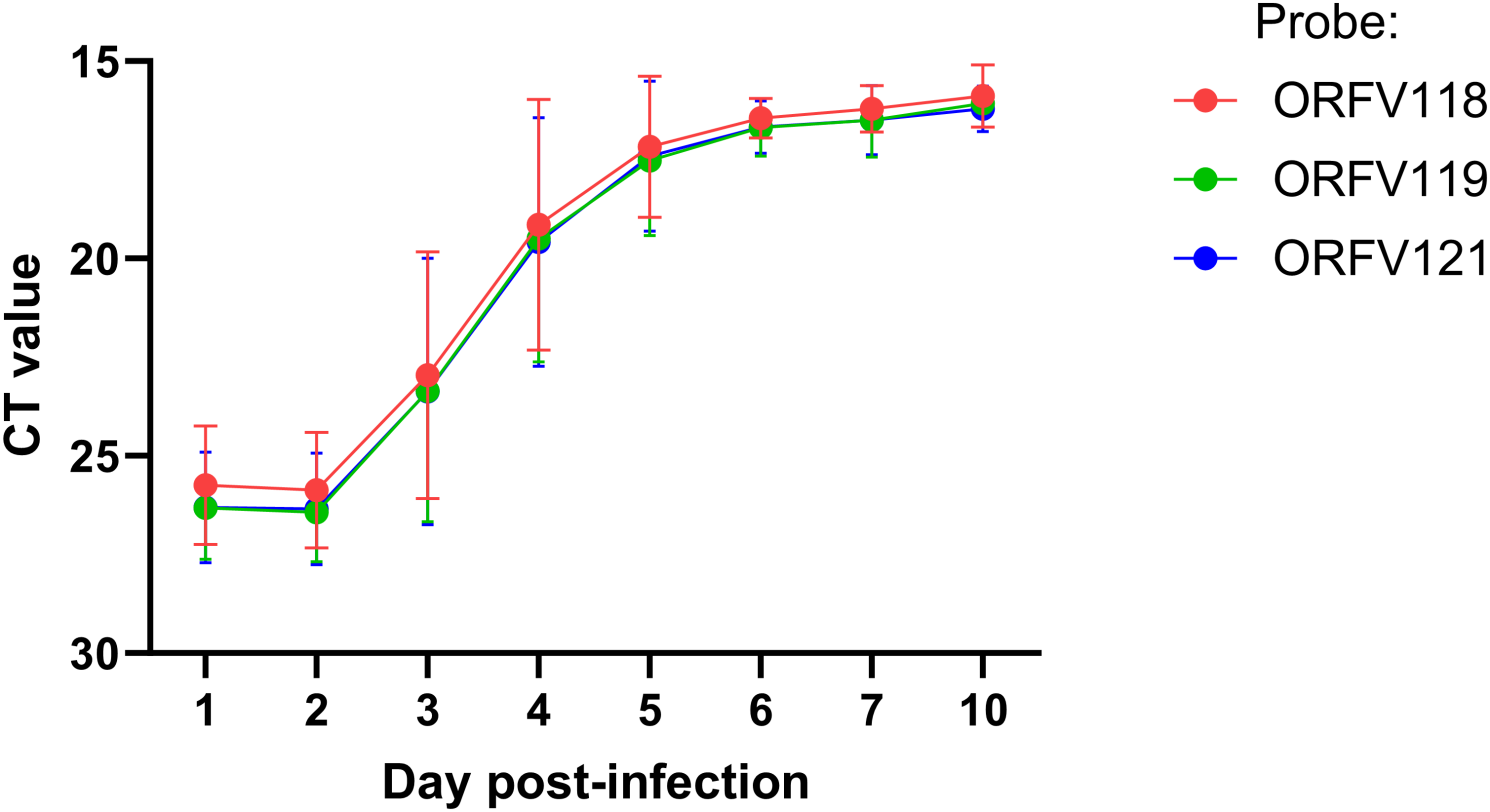
ORFV DNA quantification at lesion sites. Six sheep (#33, 190, 196, 204, 240, and 533) were inoculated with OV-IA82-RV120^3XFlag^ following full-thickness skin wounds. Punch biopsies were collected at 1 and 2 dpi (all sheep), 3 and 4 dpi (sheep # 33, 190, 204, and 533), and 5, 6, 7, and 10 dpi (sheep 190 and 204). DNA was extracted and amplified using TaqMan real-time PCR for ORFV118, ORFV119, and ORFV121. Differences between days 1-2 vs day 4 pi were significant (*p*<0.002).

Previous studies have shown that ORFV antigen cannot be detected in sheep during the first 2 days pi (eclipse period) (Osman, 1976; Jenkinson et al., 1990a), and it has been suggested that IHC might not be sensitive enough to detect the small amounts of antigen present at the early stages pi. Here, samples obtained daily for 7 days pi were processed for RNA *in situ* hybridization (RNA-ISH) using RNA probes targeting early (ORFV113) and late (ORFV011) viral genes. As negative controls, ISH signals were not observed in samples from infected sheep probed with DapB, that targets bacterial RNA sequences, and no signals for ORFV transcripts were observed for uninfected control sheep # 34 and 82 (**Figure 3B**, right panel). Importantly, keratinocytes exhibiting ISH signal for viral transcripts were not observed on multiple sections from samples from sheep # 12, 21, 30, 104, and 374 collected on days 1 and 2 pi with either viral probe. However, a small number of ISH-positive cells with dendritic morphology were observed in the dermis on days 1-4 pi (**Supplementary Figure 4**). Specific ISH signal in keratinocytes for both viral transcripts and viral DNA was observed at 60 hpi in sheep #21 and in samples from other sheep on days 3 through 7 pi. (**Figures 3A, B**). Nuclease digestion controls indicated that the positive ISH signal was mostly due to the detection of viral RNA, while viral DNA was restricted to intracytoplasmic areas likely corresponding to viral factories (**Figure 3B**, central panels). In agreement with ISH data, viral antigen was detected by IF at 60 but not 48 hpi (**Figure 3C**). When DNA from four infected wounds was assessed for ORFV DNA by qPCR, relatively small amounts of viral DNA were detected on days 1 and 2 pi. (**Figure 4**, see legend for sheep IDs). Notably, no major differences in the amount of DNA were observed between day 1 and 2 pi. This changed little on day 3pi in sheep #190, #33, and #533, while in sheep #204 a marked increase in viral DNA was observed. By day 4 pi, the increased CT values for the tested sheep with all three probes were significantly different from day 1 and day 2 pi (p<0.002). High viral DNA levels remained until day 10 pi, the last day examined (**Figure 4**). Together, data indicate that both viral transcription and DNA replication in the epidermis are prevented or blocked during days 1 and 2 pi in the host, and suggests that, in the FTW model, viral DNA replication starts around day 3 pi. The weak, mostly invariable qPCR signal detected on days 1 and 2 pi likely represents the amplification of input viral DNA.

**Figure 5:**
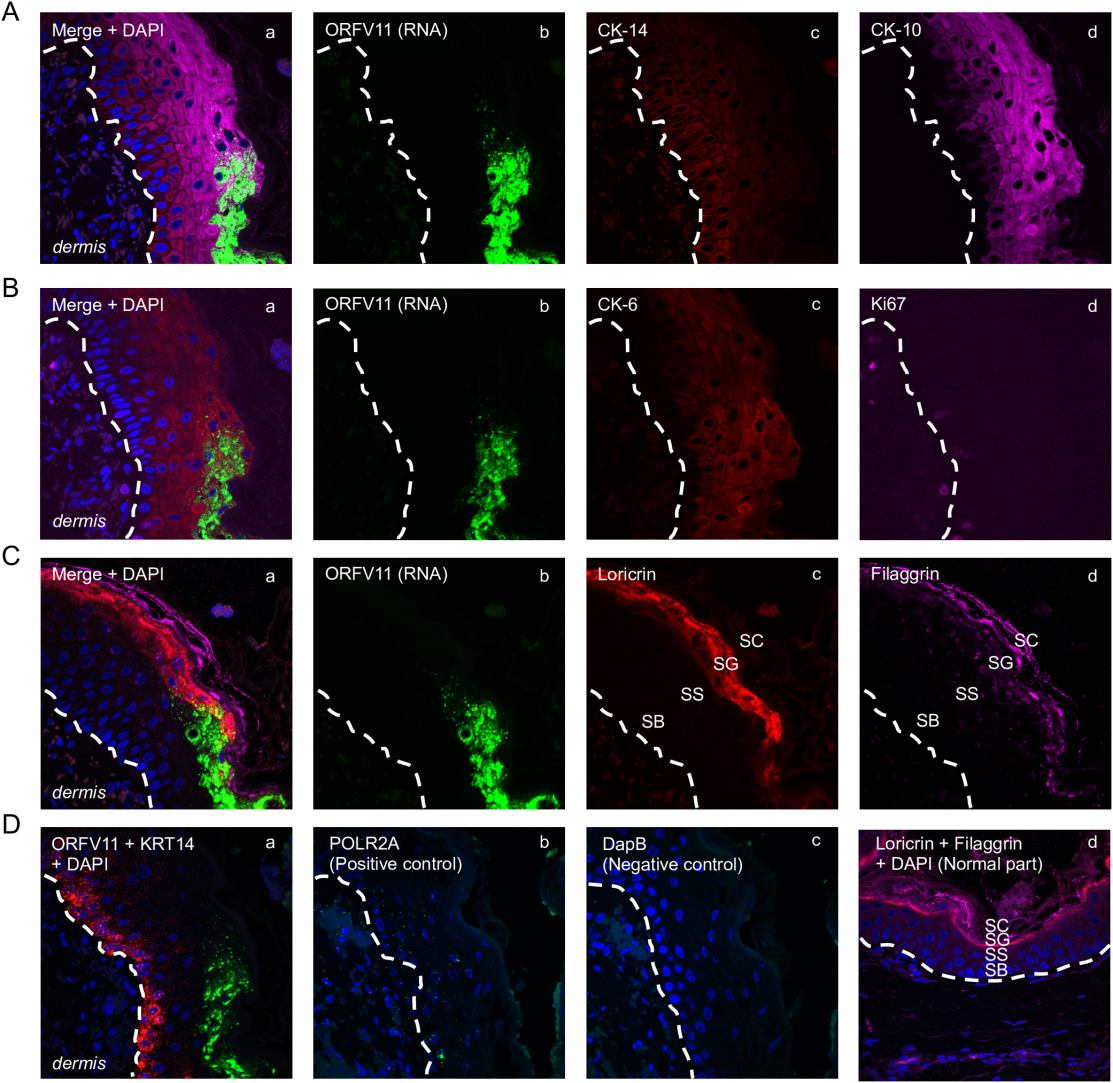
Characterization of keratinocytes supporting ORFV replication. Representative serial sections are from the 3 dpi sample from sheep #30, corresponding to **Figure 1D**’s rectangular region. **(A)** Multiplex RNA-ISH for viral RNA (green) and IF for CK-14 (red) and CK-10 (purple). **(B)** RNA-ISH for viral RNA (green) and IF for CK-6 (red) and Ki67 (purple). **(C)** RNA-ISH for viral RNA (green) and IF for Loricrin (red) and Filaggrin (purple). **(D)** a, Multiplex RNA-ISH for viral RNA (green) and cytokeratin 14 RNA (red); b, RNA-ISH for POLR2A (positive control); c, RNA-ISH for DapB (negative control); d, uninfected epidermis stained for loricrin (red) and filaggrin (purple), with DAPI. X600. Similar results with ORFV113 and ORFV011 probes.

By day 3 pi, the epidermal thickness in the PZ was of the same magnitude as for the uninfected controls (compare, for example, **Figure 5C**-a with **Figure 2**, right column), and a foci of ballooning degeneration of keratinocytes was observed around the wound margin. From here, the infection spread laterally until day 7 pi (last day sampled for microscopic studies) as mononuclear infiltration of the dermis increased. By day 5 pi, pustules and a few epidermal rete pegs developed (**Supplementary Figure 2**). By day 7 pi, however, rete pegs were approximately one order of magnitude more abundant than in the uninfected wounds at 7 dpw and became elongated and branched (**Supplementary Figure 3A**). In contrast to uninfected sheep, the epidermal closure of infected wounds was incomplete by day 7pi (**Supplementary Figure 3B**) and the leading edge was still identifiable (**Supplementary Figure 1B**), further indicating that virus infection affects epidermal behavior during healing.

### 3.3 Characterization of keratinocytes supporting ORFV replication

**Figure 6:**
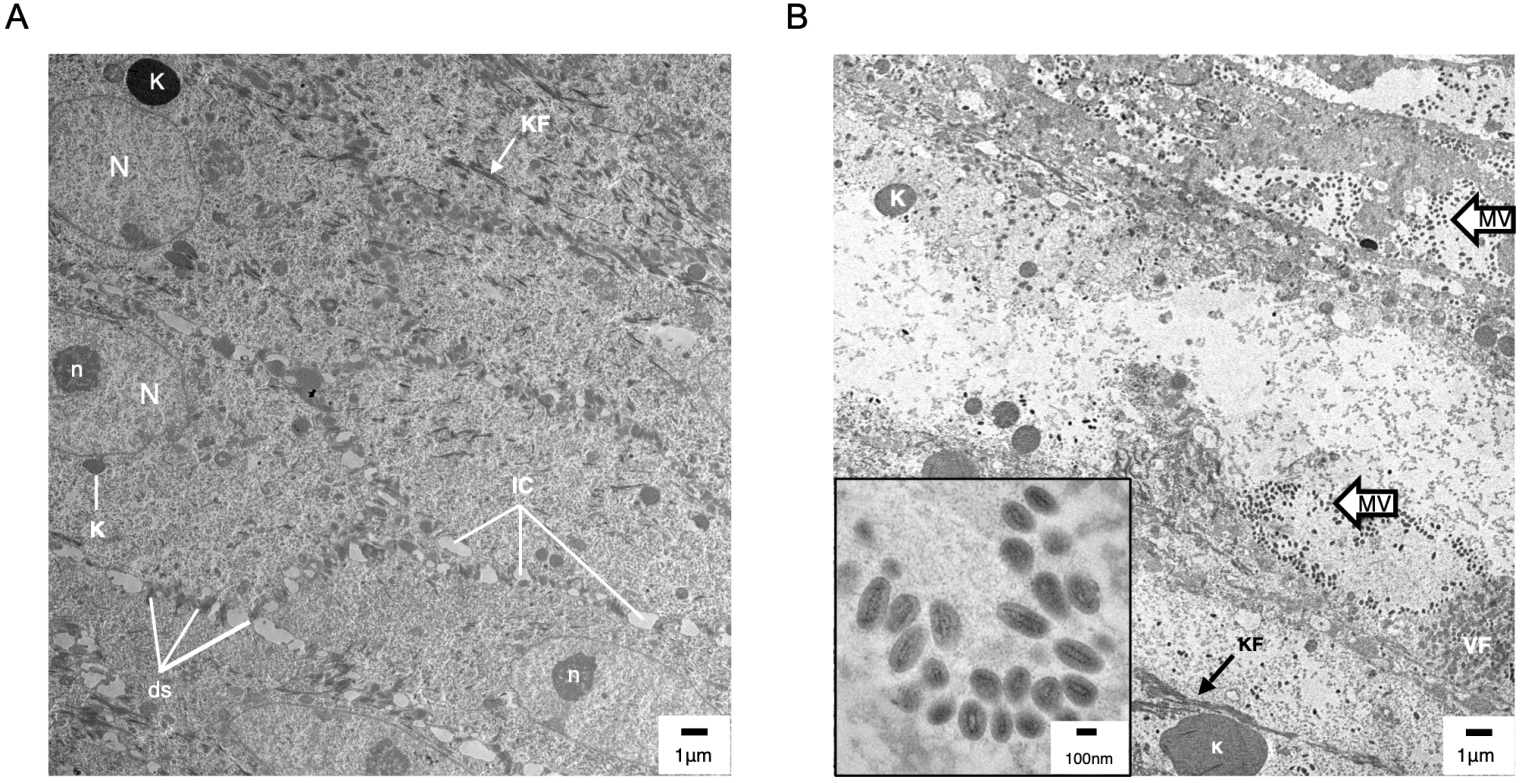
Transmission electron microscopy (TEM) of uninfected **(A)** and ORFV-infected **(B)** sheep epidermis. Following FTW, sheep were mock-infected or infected with OV-IA82-RV120^3XFlag^. Tissue samples were collected at various times pi and days pw and processed for TEM (see M&M). **(A)** Uninfected epidermis (5 dpw), SS/SG transition. N and n, keratinocyte nucleus and nucleolus, respectively; K, keratohyalin granule; KF, intermediate (keratin) filaments; ds, desmosomes; IC, intercellular space. While KF and abundant ds are found in keratinocytes of most epidermal stages, sparse and small K is a feature of the SS/SG transition. **(B)** Infected epidermis (5dpi) showing SG keratinocytes with CPE (enlargement of cytoplasm and absence of most organelles, including the nucleus) and containing clusters of mature virions (MV and inset); VF, viral factory.

The results here support previous studies showing that ORFV antigens are first detected immediately under the stratum corneum in the repair area between 2 and 3 dpi (**Figure 3A**) (McKeever et al., 1988; Jenkinson et al., 1990a). As the infection spreads laterally around the wound margin (**Figure 1B**), keratinocytes at the front of infection (FOI) show little to no cytopathic effect (CPE, i.e., ballooning degeneration) while the keratinocytes left behind show full CPE (**Figure 1D**). The latter cells, likely not viable, have been shown to lack most if not all organelles and to contain mature virions (Kluge et al., 1972). To characterize newly infected keratinocytes without interference from dying infected cells, we focused on the transition between the intact epidermis and the FOI, which encompasses the newly infected cells but excludes cells with full CPE (**Figure 1D**, rectangle). **Figures 5A–C** show serial sections through this region after multiplex RNA-ISH for viral RNA (ORFV011 probe) and double IF for CK-10/CK-14 (A), CK-6/Ki67 (B), and loricrin/filaggrin (C). Keratinocytes expressing viral transcripts (green) overlap keratinocytes that are positive for CK-10, CK-6, loricrin, and filaggrin, and negative for CK-14 and Ki67, in a region encompassing the stratum spinosum (SS)-stratum granulosum (SG) transition. The lack of CK-14 RNA expression in the area is shown in **Figure 5D**, a. Together, the results indicate that the upper PZ keratinocytes around the SS/SG transition and supporting viral replication are well into their differentiation program. These cells were found to be Ki-67- and CK-14- negative, indicating they are not proliferating cells. In support of the observations above, CPE and mature ORFV particles were only observed in the upper SS and the SG (**Figure 6**). No viral transcripts were detected in the LE during the experiment (**Supplementary Figure 1B**).

## 4 Discussion

Using a full-thickness wound model for ORFV infection, we showed that a keratinocyte subpopulation present in the PZ of the healing epidermis can support ORFV gene expression starting at 2-3 days pi in the natural host. Whether these cells are descendants of interfollicular epidermal stem cells, migrating stem cells from other epithelial niches such as hair follicles and skin glands, or both, remains to be determined (Gonzales and Fuchs, 2017). During the 7-day experiment, infected keratinocytes were confined to the PZ around the wound margins. Viral activity (CPE, antigen, and transcripts) was not observed in migrating keratinocytes of the LE.

The requirement for epidermal WHR for effective ORFV replication is noteworthy as many viruses in the *Orthopoxvirus*, *Capripoxvirus*, and *Leporipoxvirus* genera induce epidermal hyperplasia without the need for preceding skin wounds. This might be due to epidermal stimulation by a diffusible virus-encoded epidermal growth factor (EGF) homolog via EGF receptor signaling (Twardzik et al., 1985; King et al., 1986). Deletion of the two copies of the vaccinia virus EGF gene homolog VGF (vaccinia virus growth factor) resulted in a marked reduction of epidermal hyperplasia in a rabbit skin model and in virus attenuation in mice (Buller et al., 1988). PPV, including ORFV, lack an EGF-like gene (Delhon et al., 2004) and might rely on the WHR, which involves EGF induction and keratinocyte hyperplasia, for successful infection (Barrientos et al., 2008). This may partially explain the reported failure to infect sheep with ORFV by the SC and ID routes (Boughton and Hardy, 1934; McKeever et al., 1988). It has been suggested that the angiogenic ORFV-encoded vascular endothelial growth factor (vVEGF) may promote epidermal proliferation during ORFV infection. When applied to wounds, vVEGF modestly increased epidermal hyperplasia and contributed to epidermal regeneration in a mouse wound model (Wise et al., 2003, Wise et al., 2012). Deletion of the v-VEGF gene from the viral genome, however, showed no impact on epidermal hyperplasia and virus growth during the first week pi in experimentally infected sheep (Savory et al., 2000), suggesting that the epidermal changes at the PZ during the WHR suffice to promote virus replication. Indeed, we found that epidermal thickness at the PZ in infected and uninfected animals at 2- and 3-days pi (i.e., the window of time in which virus replication is first detected) were of similar magnitude, further supporting a role for the WHR in virus replication.

The results of ISH and qPCR in this study (**Figure 4** and **Figure 5**) provide strong evidence for the lack of viral gene expression and DNA replication in keratinocytes during the first 2 days after infection, thus explaining previous results showing the absence of viral antigen detection (Osman, 1976; Jenkinson et al., 1990a, Jenkinson et al., 1990b) and markedly reduced virus growth (Savory et al., 2000) during this time. While involvement of selected viral immunomodulatory factors (iMF) cannot be completely ruled out, previously characterized early ORFV iMF genes such as ORFV113 (**Figure 4A**; Khatiwada et al., 2021) and ORFV119 (Nagendraprabhu et al., 2017; not shown) were found to be transcriptionally silent by ISH. This is intriguing as PPV early gene expression and viral DNA replication are underway by 6-12 hpi in virus-infected cultured cells (Thomas et al., 1980; Balassu and Robinson, 1987; Wood and McInnes, 2003; Joshi et al., 2023). The delay in ORFV replication observed *in vivo* might be due to a low effective virus dose at the skin inoculation sites, the presence in the epidermis of mechanisms that actively prevent viral gene expression, or the lack of a favorable cellular milieu for viral gene expression (McKeever et al., 1988; Jenkinson et al., 1990a) during the first 2 days pi. The status and intracellular location of ORFV during the eclipse period remain unknown. In a type of poxvirus, vaccinia virus (VACV), viral particles are partially disassembled within minutes after entry into the cell to release transcriptionally active cores in the cytoplasm (Kates and Beeson, 1970; Pedersen et al., 2000). Cores are then fully uncoated in the proximity of ER membranes, and released viral genomes become replicative sites that contribute to viral factories (Mallardo et al., 2002). Whether ORFV is maintained as full particles in membrane-bound intracellular structures as shown early after infection in fetal lamb fibroblasts (Onwuka et al., 1995) or undergoes some degree of uncoating in keratinocytes *in vivo* during the eclipse period remains unknown. We have attempted to visualize intracellular inoculum virions and cores, and ultrastructural cellular changes in the epidermis during the eclipse phase (6, 24, 36, and 48 hpi) by TEM, but to no avail. This might be due to the sections missing the critical structures (a recurrent issue when dealing with ultrathin sections) or to eclipse virus persisting in an unknown particulate form. Regardless, it is assumed that as keratinocytes divide as part of the WHR, internalized dormant virus is segregated into daughter cells that, upon reaching a certain differentiation stage, support virus replication (Jenkinson et al., 1990a). Here, we found that a subapical population of PZ keratinocytes at the SS/SG transition supports viral gene expression starting between 2 and 3dpi. These cells express CK-10, CK-6, filaggrin, and loricrin but not CK-14 and Ki-67, indicating that they are engaged in advanced keratinocyte differentiation rather than proliferation. Bringing to light what attributes of these cells are responsible for triggering virus gene expression in the healing epidermis will require further investigation. Future studies could utilize advanced spatial molecular technologies to identify host factors and pathways that facilitate ORFV replication in the keratinocyte population identified here and to explore molecular and immune responses during the eclipse phase. Furthermore, determining whether events similar to those described here for ORFV also occur during infection by other parapoxviruses, such as bovine papular stomatitis virus (BPSV) and pseudocowpox virus (PCPV), will require the development of suitable *in vivo* infection models in their natural hosts (cattle). The observation in the dermis of cells with dendritic morphology positive for ORFV transcripts between 1 and 5 dpi is intriguing (**Supplementary Figure 4**). Although no further characterization was attempted here, it is tempting to speculate they represent infected Langerhans’ cells leaving the epidermis. MHC class II+ dendritic cells accumulate early in orf lesions (Jenkinson et al., 1991) and Langerhans cells have been shown to migrate to the afferent lymph of sheep between days 3 and 5 pi, which corresponds to the time we observed ISH-positive cells with dendritic morphology in our samples (Yirrell et al., 1991). Whether infection of these cells is productive or abortive, or whether they play a role in orf pathogenesis, including virus persistence, remains to be determined.

## Data Availability

The original contributions presented in the study are included in the article/**Supplementary Material**. Further inquiries can be directed to the corresponding author.
