# Supplementary material for: Temporal and spatial characterization of keratinocytes supporting orf virus replication
Source: Front Cell Infect Microbiol. 2025 Jan 31;14:1486778. doi: 10.3389/fcimb.2024.1486778 (PMC11825470; doi:10.3389/fcimb.2024.1486778)
Supplement: Supplementary file 1 [file Table1.docx]

Supplementary Table 1. List of antibodies and their corresponding dilutions.

| Antibody | Product Name | Host, Isotype | Application, dilution | Manufacturer, Catalog number | Secondary antibodies |
| --- | --- | --- | --- | --- | --- |
| CK10 | Cytokeratin 10 Monoclonal Antibody (DE-K10) | Mouse, IgG1 | IHC, 1:200  RNA-ISH with IF, 1:100 | Invitrogen, MA5-13705 | IHC: ImmPRESS® HRP Goat Anti-Mouse IgG Polymer Detection Kit (Vector Laboratories; MP-7452)  IF: Goat anti-Mouse IgG1 Cross-Adsorbed Secondary Antibody, Alexa Fluor™ 647 (Invitrogen, A21240) |
| CK14 | Novocastra Liquid  Mouse Monoclonal Antibody  Cytokeratin 14 | Mouse, IgG3 | IHC, 1:200  RNA-ISH with IF, 1:100 | Leica, NCL-L-LL002 | IHC: ImmPRESS® HRP Goat Anti-Mouse IgG Polymer Detection Kit (Vector Laboratories; MP-7452)  IF: Goat anti-Mouse IgG3 Cross-Adsorbed Secondary Antibody, Alexa Fluor™ 594 (Invitrogen, A21155) |
| Filaggrin | Anti-Filaggrin antibody [SPM181] | Mouse, IgG1 | IHC, 1:200  RNA-ISH with IF, 1:50 | Abcam, ab218863 | IHC: ImmPRESS® HRP Goat Anti-Mouse IgG Polymer Detection Kit (Vector Laboratories; MP-7452)  IF: Goat anti-Mouse IgG1 Cross-Adsorbed Secondary Antibody, Alexa Fluor™ 647 (Invitrogen, A21240) |
| Loricrin | Anti-Loricrin antibody [EPR7149(2)] - C-terminal | Rabbit, IgG | IHC, 1:400  RNA-ISH with IF, 1:200 | Abcam, ab198994 | IHC: ImmPRESS® HRP Goat Anti-Rabbit IgG Polymer Detection Kit (Vector Laboratories; MP-7451)  IF: Goat anti-Rabbit IgG (H+L) Cross-Adsorbed Secondary Antibody, Alexa Fluor™ 594 (Invitrogen, A11012) |
| Ki67 | Novocastra Liquid  Mouse Monoclonal Antibody  Ki67 Antigen | Mouse, IgG1 | IHC, 1:100  RNA-ISH with IF, 1:50 | Leica, NCL-L-Ki67-MM1 | IHC: ImmPRESS® HRP Goat Anti-Mouse IgG Polymer Detection Kit (Vector Laboratories; MP-7452)  IF: Goat anti-Mouse IgG1 Cross-Adsorbed Secondary Antibody, Alexa Fluor™ 647 (Invitrogen, A21240) |
| CK6 | Monoclonal Mouse anti‑Human KRT6 / CK6 / Cytokeratin 6 Antibody | Mouse, IgG2 | RNA-ISH with IF, 1:500 | LSBio, LS-C392262-20 | IF: Goat anti-Mouse IgG2a Cross-Adsorbed Secondary Antibody, Alexa Fluor™ 594 (Invitrogen, A21135) |
| FLAG | Monoclonal ANTI-FLAG® M2 antibody produced in mouse | Mouse | IF, 1:500 | Sigma-Aldrich, F1804 | IF: Goat anti-Mouse IgG (H+L) Highly Cross-Adsorbed Secondary Antibody, Alexa Fluor™ 488 (Invitrogen, A11029) |

Supplementary Table 2. Primers and probes for ORFV118, ORFV119, and ORFV121 gene used in TaqMan real-time PCR assay.

|  | Forward primer (5′→3′) | Reverse primer (5′→3′) | Probe |
| --- | --- | --- | --- |
| ORFV118 | GGCTCCATGTCATTTCAACG | GAAATCATTCTGCAACGCCTC | 56-FAM/TCGCAGGCTGGATGCTCCG/36-TAMSp |
| ORFV119 | TGGACTCTCGTAGGCTCG | CGATGCTGGCGATGAGAG | 56-FAM/CGGCGCTGTGTCATGCTGG/36-TAMSp |
| ORFV121 | CCATGAACGACTGCCTGATG | CGCTCAGCTGTGTCTCTATATC | 56-FAM/TCGTCTTGGAGGTTGGGTCTGC/36-TAMSp |
