# Supplementary material for: Temporal and spatial characterization of keratinocytes supporting orf virus replication
Source: Front Cell Infect Microbiol. 2025 Jan 31;14:1486778. doi: 10.3389/fcimb.2024.1486778 (PMC11825470; doi:10.3389/fcimb.2024.1486778)
Supplement: Supplementary file 2 [file DataSheet1.pdf]

A

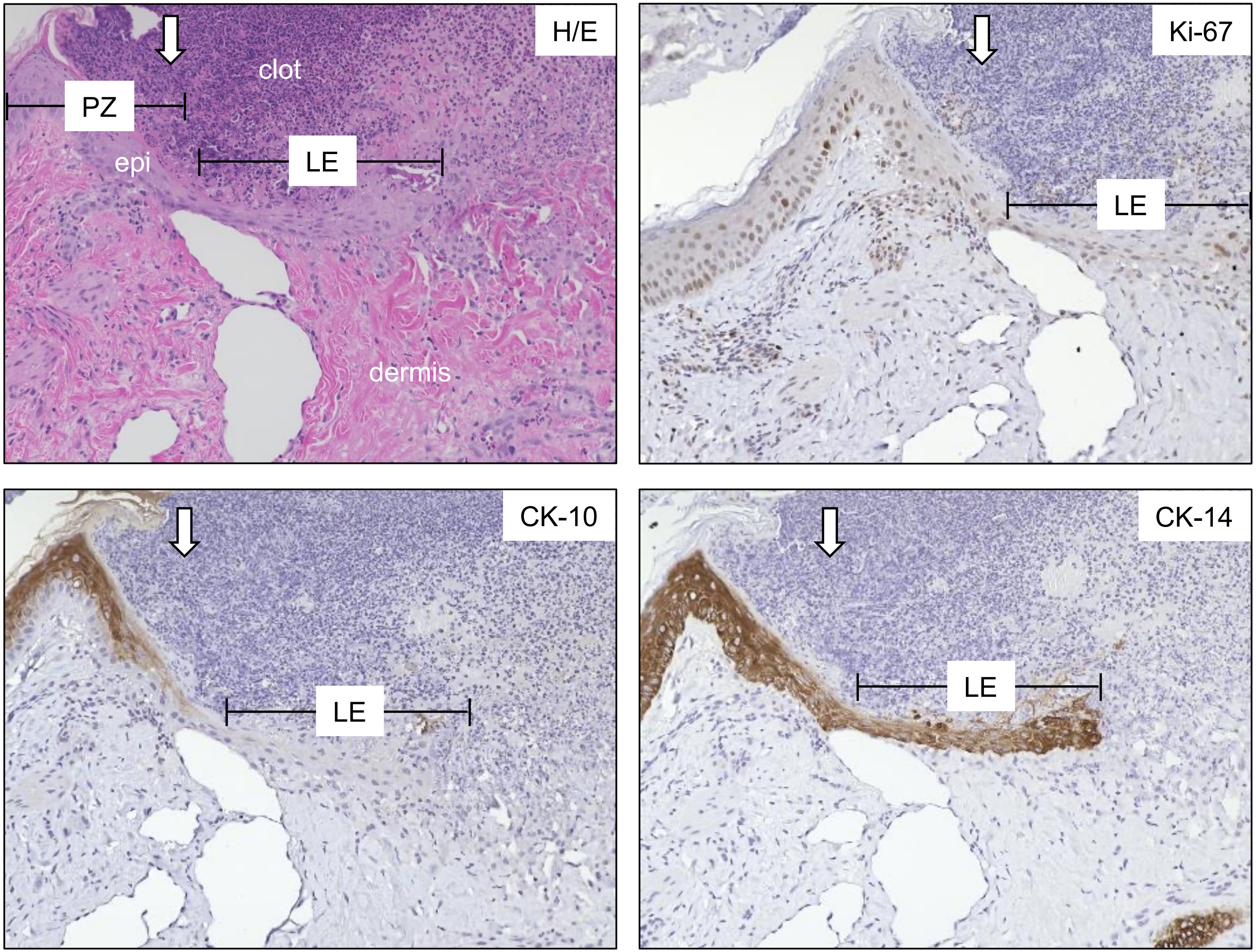

Supplementary Figure 1A. IHC characterization of wounded, uninfected epidermis. H/E staining and IHC for the indicated markers was performed on serial sections of sheep #34 and #82 samples at 60 hpi (#82 images are shown). The arrows indicate the left wound margin; PZ, proliferative zone; LE, leading edge; epi, epidermis. X200.

B

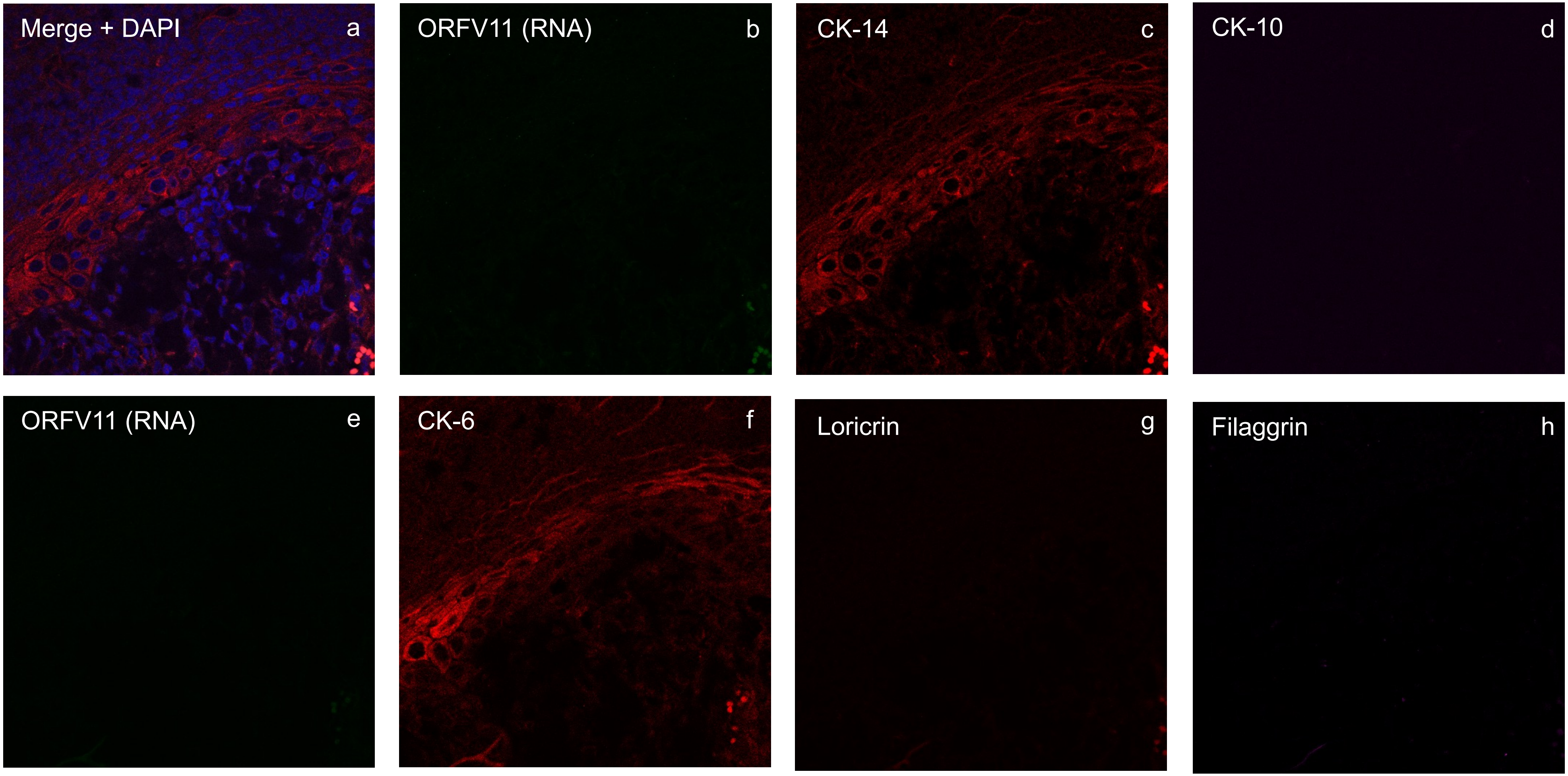

Supplementary Figure 1B. Confocal microscopy characterization of the leading edge at 7 dpi. Multiplex RNA-ISH for viral RNA (ORFV011 probe) and double IF for CK-10 and CK-14 (a-d). The leading edge is stained by antibodies for CK-14 (c, red) but not by antibodies against CK-10 (d). Multiplex RNA-ISH for ORFV011 viral RNA and combined IF for CK-6 or loricrin and Filaggrin (e-h). The leading edge is positive for CK-6 (f, red) and negative for loricrin (g) and filaggrin (h). No transcription of ORFV011 RNA was detected (e). X200.

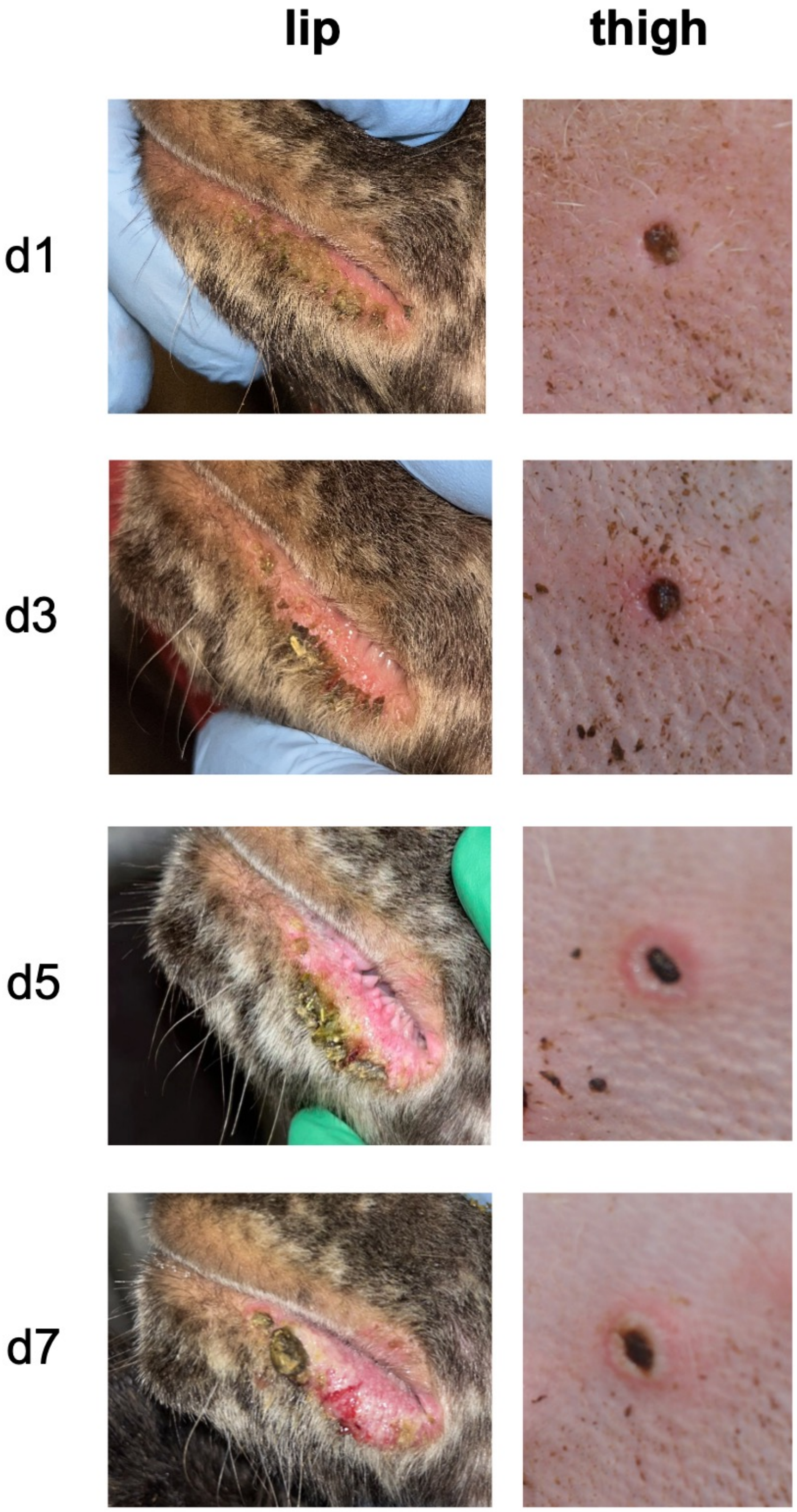

Supplementary Figure 2. **Lesion progression after experimental ORFV inoculation**. Sheep were inoculated with ORFV after skin scarification on the ventral lip near the left labial commissure (left column) and following a 3mm punch biopsy on the skin of the inner side of the thigh (right column). Shown are images on days 1, 3, 5, and 7 pi (d1-d7). Erythema around the inoculation sites is seen on d3, d5 and d7. On d5 and d7, pustules and scabs are seen in the lip, while annular pustules surrounding central scabs are seen in the leg.

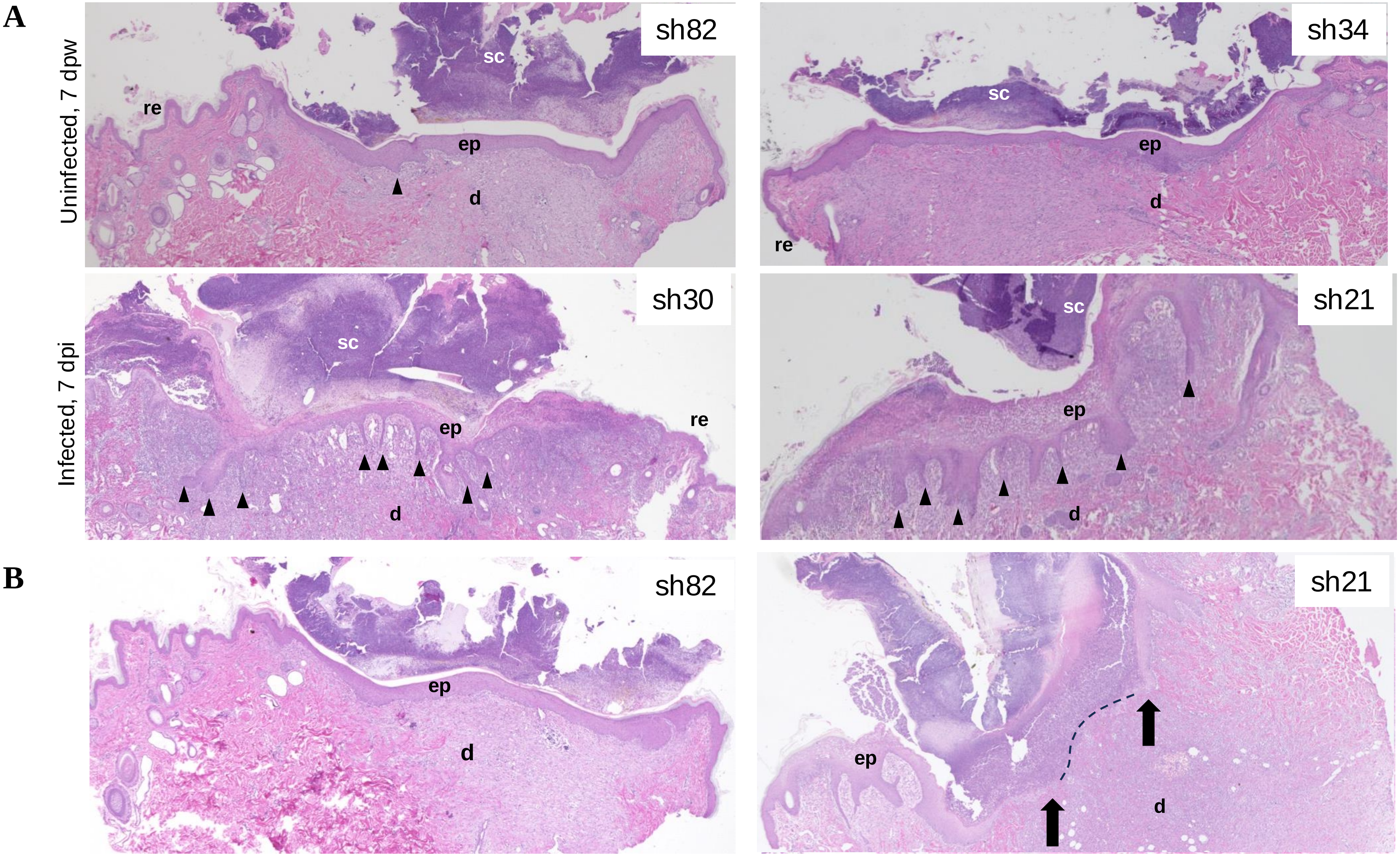

Supplementary Figure 3. **Epidermal differences between uninfected vs ORFV-infected FTW at day 7pw/pi.** A. Representative images of uninfected (top, sheep #82 and #34) and infected (bottom, sheep #30 and #21) wounds. Note the well-developed epidermal rete pegs in infected wounds (arrowheads). B. Left, complete epidermal closure in uninfected wound (sheep 82). Right, incomplete epidermal closure (sheep 21; the dashed line indicates the position of exposed dermis while the arrows indicate epidermal ends). H/E, X100. ep, epidermis; d, dermis; sc, scab; re, resting skin.

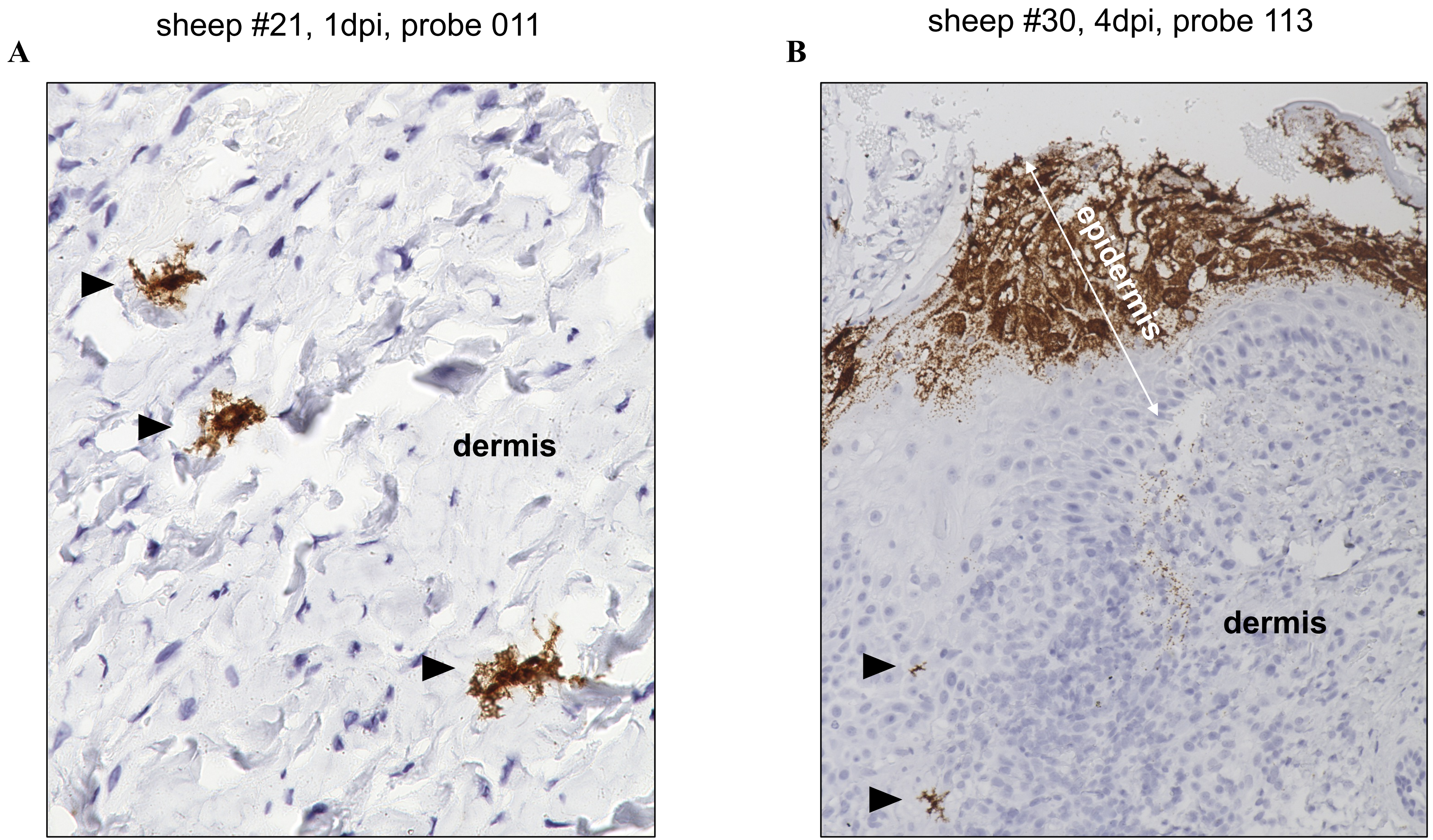

Supplementary Figure 4. ORFV transcripts in dermal cells with dendritic morphology. ISH-RNA for viral transcripts on day 1 (A) and 4 (B) pi. Positive dendritic cells are indicated by the arrowheads. Similar images were obtained with ORFV113 and ORFV011 probes. A, X600; B, X200.
